# Coexistence of diffuse large B-cell lymphoma and papillary thyroid carcinoma in a patient affected by Hashimoto's thyroiditis

**DOI:** 10.1590/2359-3997000000313

**Published:** 2017-12-01

**Authors:** Maria Trovato, Giuseppe Giuffrida, Antonino Seminara, Simone Fogliani, Vittorio Cavallari, Rosaria Maddalena Ruggeri, Alfredo Campennì

**Affiliations:** 1 University of Messina Department of Clinical and Experimental Medicine Unit of Endocrinology Messina Italy Department of Clinical and Experimental Medicine, Unit of Endocrinology, University of Messina, Messina, Italy; 2 University of Messina Department of Human Pathology Messina Italy Department of Human Pathology, University of Messina, Messina, Italy; 3 Azienda Sanitaria Provinciale Messina Italy Azienda Sanitaria Provinciale, Messina, Italy; 4 Hospital of Milazzo Unit of Radiology Messina Italy Unit of Radiology, Hospital of Milazzo, Messina, Italy; 5 University of Messina Department of Biomedical Sciences and Morphological and Functional Images Unit of Nuclear Medicine Messina Italy Department of Biomedical Sciences and Morphological and Functional Images, Unit of Nuclear Medicine, University of Messina, Messina, Italy

## Abstract

Papillary thyroid carcinoma (PTC) is the most common type of thyroid cancer. On the contrary, primary thyroid lymphoma (PTL) is a rare disease, accounting for 2% to 5% of all thyroid malignancies. Despite several cases in which both PTC and PTL arise in the setting of Hashimoto's thyroiditis (HT), the coexistence of both tumors in HT patients is very rare. Herein we report the case of a 66-year-old woman with long-standing nodular HT under replacement therapy, who presented with a fast, painless enlargement in the right anterior side of the neck. Thyroid ultrasonography demonstrated increased growth of a hypoechoic nodule in the right lobe measuring 32 × 20 mm. A total thyroidectomy was performed, and histology revealed a diffuse large B-cell lymphoma (DLBCL) on a background of florid HT. Moreover, a unifocal papillary microcarcinoma, classical variant (7 mm, pT1aNxMx), was discovered. The patient was then treated with chemotherapy for the PTL, but she did not undergo radioactive iodine ablation treatment for the microPTC as per guidelines. Two years after surgery, the patient had no evidence of recurrence of either malignancy. This rare case highlights the importance of monitoring HT patients with nodular lesions, especially if they have long-standing disease. In addition, PTL should be considered for differential diagnosis in elder HT patients who present with sudden thyroid enlargement.

## INTRODUCTION

Papillary thyroid carcinoma (PTC) is the most common type of thyroid cancer, and its incidence has been increasing in the last few decades, with a large prevalence of small tumors (1). The occurrence of PTC in patients affected by Hashimoto's thyroiditis (HT) is a well-known event reported by literature (2): a long-standing HT could lead to high TSH levels, thus becoming a growth factor for malignancies, but on the other hand HT could be a sort of protective agent against the aggressiveness of PTC. In fact, in HT patients the neoplasia is generally discovered at a younger age; it is characterized by smaller nodules and a less advanced TNM stage, without local or systemic invasion (3,4). Obviously, even other factors could contribute to a better prognosis, such as the more frequent ultrasonographic controls that HT patients undergo, so as to allow an early diagnosis of malignancy. Conversely, primary thyroid lymphoma (PTL) is a rare disease that accounts for 5% of all thyroid tumors: in 70% of cases it appears as diffuse large B-cell lymphoma (DLBCL), which usually presents with a more aggressive course and has a worse outcome. The other 30% comprises mucosa associated lymphoid tissue (MALT) lymphomas, which are indolent in most cases and have a better response to systemic treatment (5,6). Some cases of association between HT and both PTC and MALT lymphomas have been described (7-9), while a DLBCL in the context of coexisting HT and PTC is very rare (10).

## PATIENT

**Figure 1:**
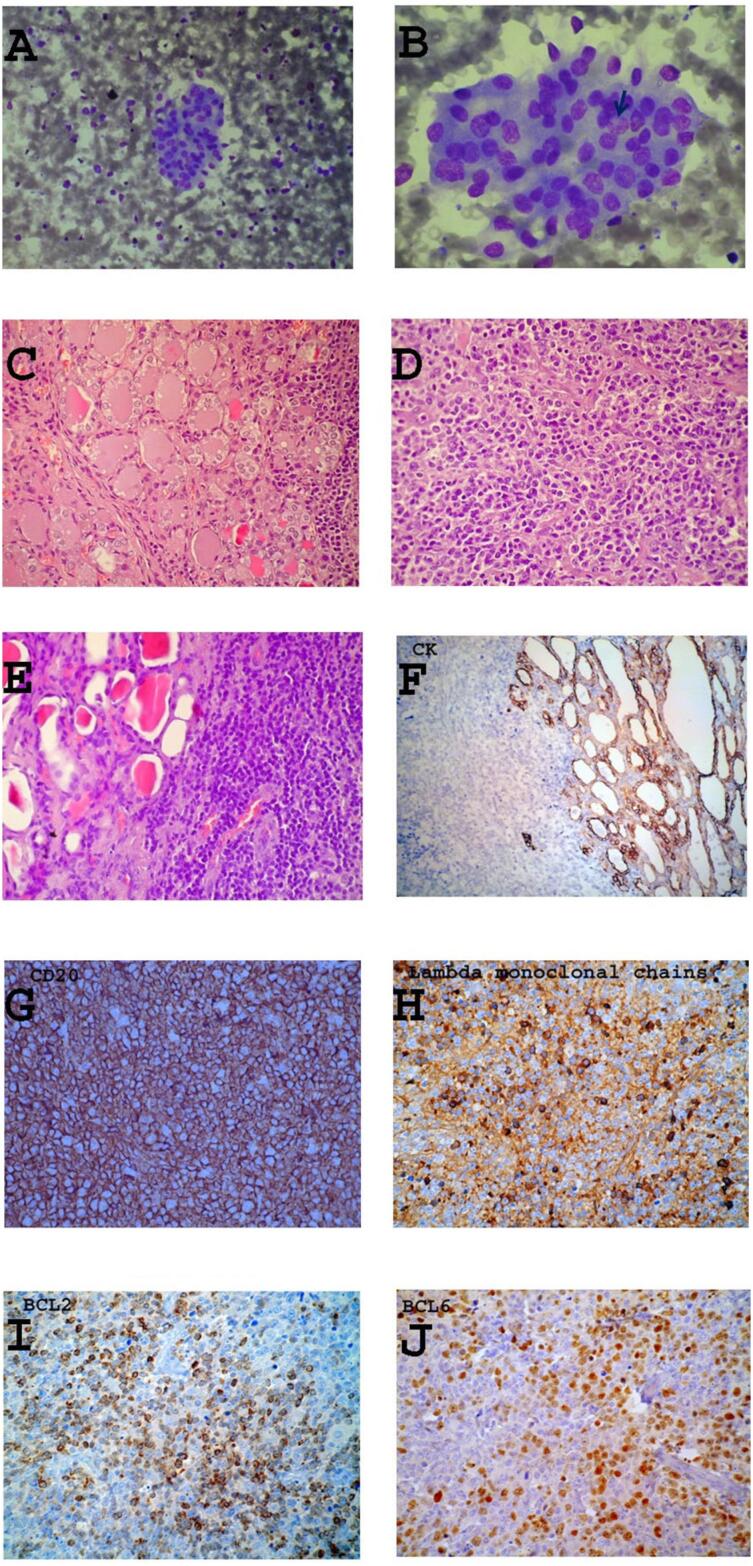
Cytological (panels AB) and histological (panels CE) features of papillary thyroid microcarcinoma and diffuse large B-cell lymphoma occurring in the context of HT. Immunohistochemical characterization of diffuse large B-cell lymphoma (panels FJ). **Panel A:** Moderate amount of lymphocytes, filamentous cell debris, and a cellular formation composed by polygonal follicular cells (May-Grünwald Giemsa, MGG; total magnification x100). **Panel B:** High magnification highlights the presence of areas of moderate anisokaryosis in a cluster of follicular cells; one cell presents also a small intranuclear inclusion (black arrow) (MGG, total magnification x400). **Panel C:** Unifocal papillary microcarcinoma (7 mm in size, pT1aNxMx) with no aggressive histology (hematoxylin and eosin, H&E; total magnification x200). **Panel D:** Diffuse large B-cell lymphoma (DLBLC), Stage 1A (H&E, total magnification x200). **Panel E:** Hashimoto's thyroiditis associated with parenchimatous goiter (H&E, total magnification x200). **Panels F, G, H, I, and J:** Immunoreactions for cytokeratin, CD20, lambda monoclonal chains, BCL2 and BCL6, respectively (F and G, total magnification x200; G, H, and I, total magnification x400).

Herein we describe the case of a 66-year-old woman, with no family history of thyroid cancer, affected by HT under replacement therapy with L-thyroxine (125 micrograms per day) and long-standing nodular goiter. She was referred to our division for a recent painless enlargement in the right anterior side of the neck, complaining of mild and intermittent dysphagia to solid and liquid foods; she denied any voice change or dyspnea, while physical examination demonstrated an enlarged thyroid gland with a palpable, firm nodular lesion in the right lobe, moving with deglutition. Thyroid ultrasonography (US) demonstrated the growth of a hypoechoic nodule in the right lobe, measuring 32 × 20 mm (the maximum diameter was 18 mm at the previous control, almost 12 months before). US-guided fine needle aspiration biopsy (FNAB) of the right-sided nodule and cytological examination revealed atypical epithelial cells and lymphocytic infiltration, concluding for indeterminate lesion (THYR3). The patient was referred to total thyroidectomy. Histology was compatible with a DLBCL, revealing large atypical lymphocytes with irregular nuclei, condensed chromatin and small nucleoli on a background of florid HT, characterized by lymphocytic aggregates with germinal centers and thyroid follicles of various sizes with dense colloid and Hurtle cell changes (Figure 1). Immunohistochemistry of the atypical lymphocytes confirmed CD20, anti-BCL2 and anti-BCL6 positivity, with monoclonal lambda chains, so the neoplasia was staged as 1A. Moreover, a unifocal papillary microcarcinoma, classic variant (7 mm, pT1aNxMx), was discovered. Staging studies for the PTL were performed, including total-body CT and bone marrow biopsy, showing no evidence of systemic disease or metastases. The patient was then treated with chemotherapy for the PTL, while she did not undergo radioactive iodine ablation treatment for the micro- PTC as per guidelines (11). At the last control in our hospital, two years after surgery, the patient had no evidence of recurrence of either malignancy.

## DISCUSSION

In patients affected by HT, thyroid architecture is usually altered by the chronic inflammation, often presenting so-called “pseudo-nodules”; nonetheless, the presence of real nodules is also a common finding in our clinical practice, especially in iodine-deficient areas (12,13). The histology of these lesions is widely variable, ranging from nodules with benign features to malignant ones, deriving from thyroid cells or lymphocytes. PTC is the most frequent thyroid tumor, and several studies have shown a significant association with HT (2), which could be explained with the progressive rise in TSH levels caused by long-standing thyroiditis; in fact, TSH is a known growth factor for thyroid nodules. In a recently published prospective study, TSH ≥ 1 μIU/ml was an independent predictor of thyroid cancer together with antithyroglobulin antibodies (TgAb), whose role is actually unknown: the authors suggest a possible, specific tumorigenic inflammatory response (14). In addition, in a study by Wirtschafter and cols., mRNA expression for the RET/ PTC 1 and RET/PTC 3 oncogenes was found even in a large number of HT patients without PTC, showing molecular genetic evidence of cancer in this population and thus highlighting their higher risk of developing a clinically expressive disease (15). However, most of the data from the literature do not clearly demonstrate a causal relationship between the two conditions, and it also has been hypothesized that the progressive increase of serum TSH in HT patients, rather than autoimmune thyroiditis per se, may play a major role in the association of PTC with HT (16,17). On the other hand, several papers report a better prognosis for PTC if it co-occurs with HT, which somehow could play a protective role against the aggressiveness of neoplasia: thyroid peroxidase antibodies (TPOAb) could probably drive a cytotoxic response against the inflammation (2-4,14). HT, however, is also a risk factor for PTL, as cellular changes due to chronic antigenic stimulation could evolve to malignancy, as demonstrated by the finding of clonal B cells, generally present in lymphomas, in HT patients (18). The onset of PTL generally happens many years after the diagnosis of HT, but Watanabe and cols. reported some cases of PTL discovered on average 18 months after the diagnosis of HT (19). In a unifying pathogenetic hypothesis, in such patients autoimmunity may exert tumorigenic actions in two ways: first, because of the chronic antigenic stimulation lymphocytes could gradually gain monoclonality for heavy chains with progression toward DLBCL; second, inflammation could produce tumorigenic compounds, e.g., cyclooxygenase-2, which has been detected in thyroid epithelial neoplasms and HT (20). At the same time, some protective factors could interfere with the tumor. In particular, as in Yamakawa and cols., the activation of the complement system, usually higher in HT patients, could prevent the lysis of neoplastic cells, thus explaining the chances of a better outcome (21). In the case we describe, we detected two distinct and simultaneous malignancies, PTC and PTL (this one as DLBCL), originating, respectively, from follicular cells and lymphocytes. To the best of our knowledge, this is the second patient in which a DLBCL had been reported in the context of Hashimoto's thyroiditis in association with PTC (10). Of note, a DLBCL has a more aggressive course and a worse prognosis compared to MALT lymphomas. Data concerning the optimal management of such an association of neoplastic diseases are scanty in the literature. In most cases of PTL, however, the diagnosis is made after a rapid growth of a thyroid-related mass often associated with compressive symptoms, e.g., hoarseness or dyspnea, and the patients are referred to surgery. The management of one does not affect the management of the other neoplasm (7); the prognosis, too, does not seem to be worsened by the coexistence of the two diseases, but rather is more likely affected by the one having the worse stage (7-9). Thus, when PTC and PTL coexist in the same patient, a careful staging of both diseases is mandatory, and treatment has to prioritize the tumor with the worse prognosis and/or the worse stage at diagnosis.

In conclusion, considering the higher risk of neoplasia in HT patients with long-standing disease and nodular lesions, our case highlights the importance of regular follow-up in order to reach an early diagnosis: in particular, a sudden thyroid enlargement in elder patients with HT should lead physicians to consider PTL in the differential diagnosis.
